# Electrocardiographic and Electrooculographic Responses to External Emotions and Their Transitions in Bipolar I and II Disorders

**DOI:** 10.3390/ijerph15050884

**Published:** 2018-04-28

**Authors:** Guorong Ma, Chu Wang, Yanli Jia, Jiawei Wang, Bingren Zhang, Chanchan Shen, Hongying Fan, Bing Pan, Wei Wang

**Affiliations:** 1 Department of Clinical Psychology and Psychiatry/School of Public Health, Zhejiang University College of Medicine, Hangzhou 310058, China; guorong_ma@aliyun.com (C.M.); wangchu17@zju.edu.cn (C.W.); medicojia@163.com (Y.J.); wjwei513@126.com (J.W.); bingrenz@zju.edu.cn (B.Z.); ccshen@zju.edu.cn (C.S.); fan_hongying@yeah.net (H.F.); 2 Department of Psychology and Behavioral Sciences, Zhejiang University College of Science, Hangzhou 310058, China; 3 Department of Psychiatry, Second Affiliated Hospital, Zhejiang University College of Medicine, Hangzhou 310058, China; icygirl2006@126.com

**Keywords:** affective state, bipolar I and II disorders, electrocardiogram, electrooculogram, emotional stimulation

## Abstract

Bipolar disorder has two main types, bipolar I (BD I) and II (BD II), which present different affective states and personality characteristics, they might present different modes of emotional regulation. We hypothesized that the electrocardiogram and electrooculogram to external emotions are different in BD I and BD II. We asked 69 BD I and 54 BD II patients, and 139 healthy volunteers to undergo these tests in response to disgust, erotica, fear, happiness, neutral, and sadness, and their transitions. Their affective states were also measured. The heart rate in BD I was significantly higher under background fear after target neutral. The eyeball movement was quicker in BD I under target happiness after background disgust; in BD I under target sadness after background disgust; and in BD I under background disgust after target neutral. Some electrocardiographic and electrooculographic changes were correlated with affective states in patients. BD I and BD II had different physiological responses to external emotions and their transitions, indicating different pathophysiologies and suggesting different emotional-therapies for BD I and BD II.

## 1. Introduction

Emotion plays a vital role in our lives, and inability to regulate or inhibit emotional responses efficiently contributes to various psychiatric disorders [1]. Bipolar disorder, which has two main types—bipolar I (BD I) and II (BD II)—is particularly characterized by emotional variations and difficulties in emotion regulation [2,3]. Besides the high risk of suicide, bipolar disorder also displays public health or public safety significance [4,5,6], for instance, with an increased risk of offending in every category of justice involvement [7]. In clinics, BD I presents mania and depression and has a higher prevalence of reckless activity, distractibility, and increased self-esteem [8], while BD II presents hypomania and depression and has higher episode frequency and comorbidity, more suicidal behavior, and rapid cycling [9]. Clinical managements of BD I and BD II are similar, but studies have shown that these patients responded differently to medication or other non-pharmacological treatments [10,11]. Investigators thus are looking for more evidence regarding the different pathophysiologies of BD I and BD II, especially those relating to emotional processing [12].

In healthy individuals, processing of an emotional stimulus includes an early perception, and a later response which includes emotional expressing and regulating [13]. The underlying mechanisms of these stages include attentional and cognitive resource mobilization and behavioral activation [14]. In different psychiatric disorders (e.g., BD I and BD II), the patterns of recognition and response to external emotion might be different. For instance, BD I had a lowered emotional recognition and tended to interpret sadness as fear [15]. BD I also showed a worsened recognition of fear, disgust [16,17], and happiness [17]. Neuroimaging techniques have shown some cerebral responsiveness when processing facial emotions in BD I and BD II patients, for instance, they displayed hippocampal over-activation to fear [18]. Compared to BD II, BD I showed more activation in dorsolateral prefrontal cortex, amygdala, and accumbens, and lower functional connectivity between the dorsolateral prefrontal cortex and amygdala in response to fear [19].

Besides the above-mentioned studies which present no clear delineation between BD I and BD II, there might be some differences regarding the peripheral responses to an external emotion between them. The outcome of bipolar disorder can be shaped by environmental variables, such as psychosocial events or external emotions [20]. For instance, positive life events predict manic symptoms of BD I [21], while negative ones such as increasing arguments with the spouse, business failure, and serious illness of a family member are significantly associated with subsequent severity of both manic/hypomanic and depressive symptoms [22]. These affective variations might eventually turn into threats to patients themselves and the public [7]. Regarding physiological measures, specifically for automatic or implicit emotional response, one candidate parameter is the heart rate (the RR interval in electrocardiogram, ECG). A heart rate increase was observed in response to anger, fear, and sadness, and a decrease to disgust [23,24]. Some scholars also reported a temporary decrease of heart rate to negative emotional-stimulation, while an unchanged one to positive stimulation [25,26]. For the voluntary or explicit emotional response, eyeball movement as reflected in the electrooculogram (EOG) is another measurement candidate. Emotional responses are associated with eyeball movement, for example, exposure to pictures evoking aggression or erotica (erotism) was associated with reduced eye fixation [27].

On the other hand, the regulation of an emotion might be accomplished by voluntary effort such as exaggerating or suppressing a response of an individual [28], or by situation selecting such as choosing or avoiding specific environments [29]. In addition, emotional transitions were shown to lead to consistently different outcomes than their corresponding steady-state emotions during social interactions [30]. Characterization of peripheral physiological responses when an individual is emerged in an external emotion or during the transition from one external emotion to another would identify more differences between BD I and BD II. When referring to the inhibitory brainstem reflex, the duration of the second exteroceptive suppression of temporalis muscle activity was more shortened under sadness in BD II than that in BD I, and the latency of the second exteroceptive suppression under erotica was more prolonged in BD I, while more prolonged under happiness and sadness in BD II [31]. When referring to the excitatory brainstem reflex, blink reflex R2 components were more delayed under disgust, erotica, fear, and happiness in BD I, and were more decreased under disgust, erotica, fear, happiness, and sadness in BD II [32]. However, to date, there has been no report about the ECG/EOG responses to different external emotions and their transitions in BD I or BD II.

Based on the above-mentioned neurophysiological changes during the external emotions in BD I and BD II, we might speculate that the peripheral physiological-responses during the external emotions and their transitions from positive to negative ones or vice versa are different in these two types of bipolar disorder. BD I patients with manic and mixed phases reported an elevated arousal level when viewing all types of pictures [33], and BD I patients with manic phase showed a worsened overall recognition of facial emotion [16]. The search for the associations between physiological measures and concurrent affective states would help to clarify phaseological characteristics in these two types of bipolar disorder. We thus hypothesized that: (1) BD I more actively reacts to positive emotions, such as erotica, happiness, or their transitions to other emotions, with an accelerated heart rate and a quicker eyeball movement; and BD II more actively reacts to negative emotions, such as disgust, fear, sadness, or their transitions to other emotions, with an accelerated heart rate and a quicker eyeball movement; and (2) the ECG and EOG changes to external emotions and their transitions are correlated with their ongoing mania/hypomania or depression. Therefore, we have invited BD I and BD II patients and healthy volunteers to undergo ECG and EOG tests to the externally positive (erotica and happiness), negative (disgust, fear, and sadness), and neutral emotions and their transitions. Participants were also invited to answer questionnaires targeting their own ongoing affective states.

## 2. Materials and Methods 

### 2.1. Participants

Altogether 267 participants were invited to attend the current study, five participants were excluded due to their inattention during the test or to their missing one or both physiological measures. Finally, there were 139 healthy volunteers (62 females and 77 males; mean age: 24.18 years with 7.78 S.D.; range: 17–59 years) who were recruited from the university, hospital staff or community; 69 outpatients with BD I (35 females and 34 males; mean age: 22.49 ± 5.92; range: 18–50), and 54 BD II (30 females and 24 males; mean age: 24.56 ± 5.93; range: 16–40) who were recruited from a psychiatric clinic. Patients were firstly interviewed by junior psychiatrists or clinicians and then diagnosed according to the Diagnostic and Statistical Manual of Mental Disorders, Fifth edition (DSM-5) [2] criteria by an experienced psychiatrist (WW). There was no significant difference when regarding either age (one-way ANOVA, main effect, F (2, 259) = 1.74, *p* = 0.18, mean square effect (MSE) = 84.38), or gender (Pearson’s chi-square with Yates correction, *χ*^2^ = 2.06, *p* = 0.36) among the three groups. All participants had to be medication-free for at least a month, were confirmed to have no other confounding factors including schizophrenia, schizoaffective disorder, nor prior history of head injury, alcohol or tobacco abuse, psychoactive substance abuse, central nervous system inflammation, nor other neurocognitive disorders through a semi-structured clinical interview. Two co-authors (G.M. and C.W.) were available to aid participants (including the hyperactive BD I patients) in the proper filling of the required demographic information, questionnaires and the informed consents, and to ensure corrective feedbacks. The study protocol was approved by a local ethics committee (no. ZGL201404-2-1) and all participants had given their written informed consents (the informed consents of the young adolescents were signed by their guardians).

### 2.2. Questionnaires

Participants were asked to complete the following three questionnaires in a quiet room.

- The Mood Disorder Questionnaire (MDQ) [34], which consists of three parts, including 13 forced-choice (yes or no) questions to assess the presence of symptoms and behaviors related to mania or hypomania, one question to determine whether two or more symptoms have been experienced at the same time, and one question to determine the extent to which symptoms have caused functional impairment on a scale ranging from “no problems” to “serious problems”. Its internal reliability was 0.79 according to a recent study in a Chinese sample [35].
- The Hypomania Checklist-32 (HCL-32) [36], which is a self-assessment instrument comprising 32 items for detecting hypomanic symptoms. Individuals were instructed to answer the forced-choice (yes or no) questions about emotions, thoughts, or behaviors, and to answer questions regarding the duration, the impact on family, social and work life, or people’s reactions. According to a recent study [37], its internal reliability was 0.88 in a Chinese sample.
- The Plutchik-van Praag Depression Inventory (PVP) [38], which consists of 34 items. Each item has three scale points (0, 1, 2) corresponding with increasing depressive tendencies. Subjects have “possible depression” if they score between 20 and 25, or “depression” if they score above 25. According to a recent study [39], the internal reliability of the inventory was 0.94 in a Chinese sample.

### 2.3. External Emotional Stimuli

The emotional stimuli were six scenes of distinct emotions (or epochs) composed of high arousal and valence pictures and sounds of the same domain. Pictures were selected from the International Affective Picture System [40] and sounds from the International Affective Digitized Sounds [41]. The six external emotional epochs were namely disgust (picture code: 9301; sound code: 700), erotica (4611; 201), fear (3001; 275), happiness (2045; 110), neutral (7175; 152), and sadness (2799; 293). In each emotional epoch, a color picture was presented horizontally (768 × 512 pixels), sustaining about 19.8° × 13.5° of visual angles. Meanwhile via headphones, a sound of 6 s in duration and 40–50 dB in intensity, was repeatedly delivered and lasted for the same time duration as for the picture. Considering that hypersexuality is frequently reported in bipolar disorder [42,43,44], and happiness and sadness are prominent affections in this disorder [2], we chose erotica, happiness, neutral, and sadness as target emotions. Each target emotion was paired with the other remaining (five) emotions individually, resulting in a total of 20 emotional combinations. Within each combination, one silent white screen (blank) was presented first for 10 s, then one emotion (background) was presented for 6 s, then another emotion (Target) was presented for 8 s, then the previous background emotion was presented again for 10 s. For the sake of brevity, designations were used for each emotional epoch within different emotional combinations (Table 1).

### 2.4. Physiological Test Procedure

After 10 min rest, participants were led to a dimly lit room and seated 100 cm in front of a computer screen. They were equipped with a headphone and were attached with electrodes. Twenty emotional combinations were presented in a randomized order for each participant. Physiological responses measured during the five seconds before presentations of each emotional combination were treated as the baseline (blank) data. During blank epochs and during the presentation of each emotional combination, participants were instructed to keep their head and body motionless. After each presentation, participants were asked to report their perceived intensity of the target emotion on a visual analogue scale (VAS) which ranged from 0 (none) to 8 (most intense).

### 2.5. Recordings

The biosignals were recorded with a two-channel bio-amplifier (Model MP150, Biopac Inc., USA) which were connected with Ag-AgCl disposable electrodes. ECG was recorded using an ECG100C amplifier and three Ag-AgCl electrodes. Two electrodes were affixed on the right arm and the left leg, and ground electrode was placed on the right leg. ECG signals were amplified using the following hardware setting: Amplifier Gain: 2000; Mode: Normal; Notch: 50 Hz; and Band-pass: 0.5–100 Hz. EOG recordings were obtained using an EOG100C amplifier, by placement of two Ag-AgC1 electrodes 1.5 cm from the outer canthus of each eye. EOG signals were amplified using the following hardware setting: Amplifier Gain: 2000; Mode: Normal; Notch: 50 Hz; and Band-pass: 0.05–100 Hz. Analog data of both ECG and EOG were sampled at 1 kHz using an MP150 analog/digital converter and recorded online with AcqKnowledge (version 4.2.0, BIOPAC Systems, Inc., Goleta, CA, USA) software for Windows.

### 2.6. Physiological Data Reduction and Normalization

**Figure 1:**
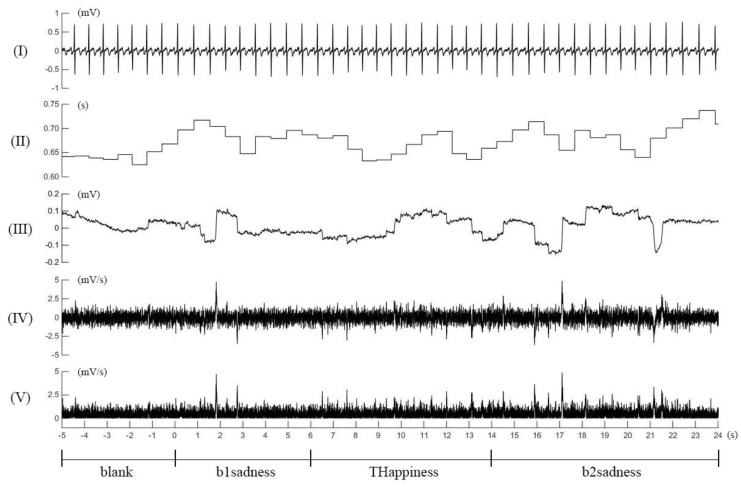
Illustrations of electrocardiogram and electrooculogram and their rectifications and quantifications in a healthy volunteer under blank-b1sadness-THappiness-b2sadness emotional combination: (**I**) electrocardiogram raw; (**II**) RR interval; (**III**) electrooculogram raw; (**IV**) dp/dt of electrooculogram; (**V**) absolute value of the (**IV**). See Table 1 for explanations of the emotional epochs.

Off-line processing of the digitized physiological data was conducted with the AcqKnowledge software. The five seconds ECG during blank before background emotion, and the first five seconds ECG during each background or target emotion epoch (see Table 1) were identified, and their respective mean RR intervals (unit:s) were calculated. The five seconds EOG during blank before background emotion, and the first five seconds EOG during each background or target emotion epoch were identified, and their EOG derivatives (dp/dt, 1000 samples/s) were calculated. The absolute values of the derivative were used to globally represent the velocity of horizontal eyeball movement [45]. Figure 1 shows examples of the rectification and quantification of the ECG and EOG signals in a healthy volunteer under the stimulation sequence of blank-b1sadness-THappiness-b2sadness.

In order to minimize the individual differences among participants in terms of their VAS scores as well as physiological responses while experiencing a specific emotion, we normalized the data acquired through quantification to [0, 1] by

*x’(t)* = [*x(t)* − min]/(max − min)

where *x’(t)* is the normalized data, *x(t)* is the original data, min is the minimum of all the original data, and max is the maximum of all the original data [46]. Normalized data were administered into further analyses.

### 2.7. Statistical Analyses

Two-way ANOVA (group X emotion) was applied to the ECG, EOG, and VAS data in the three groups of participant. One-way ANOVA was applied to the scale scores of MDQ, HCL-32, and PVP in the three groups of participant. Whenever a significant main effect was found, post hoc analysis by the Bonferroni test was employed to evaluate between-group differences. A *p*-value less than 0.05 was considered as significant for these group comparisons. The relationships between ECG/EOG data and MDQ, HCL-32, and PVP scale scores were assessed by the Pearson correlation test. In order to reduce the risk of Type I error, we took a *p*-value less than 0.01 and |*r*| ≥ 0.35 as significant regarding correlations.

## 3. Results

### 3.1. Measures of Concurent Affective States

The mean PVP scores were significantly different among the three groups of participant (F (2, 259) = 50.83, *p* < 0.001, MSE = 5165.71), with that in BD II higher than those in BD I (*p* < 0.001, 95% confidence interval (CI = 3.55~12.38) and healthy controls (*p* < 0.001, 95% CI = 11.93~19.72). The mean PVP score in BD I was also higher than that in controls (*p* < 0.001, 95% CI = 4.28~11.43). The mean MDQ scores were significantly different among the three groups of participant (F (2, 259) = 300.10, *p* < 0.001, MSE = 1065.90), with that in BD I higher than those in BD II (*p* < 0.001, 95% CI = 5.23~6.88) and controls (*p* < 0.001, 95% CI = 5.95~7.28). The mean HCL-32 scores were also significantly different among the three groups of participant (F (2, 259) = 89.44, *p* < 0.001, MSE = 1622.74), with those in BD I (*p* < 0.001, 95% CI = 6.30~9.32) and BD II (*p* < 0.001, 95% CI = 4.12~7.41) higher than that in controls, and the score in BD I higher than that in BD II (*p* = 0.03, 95% CI = 0.19~3.92) (Table 2).

### 3.2. Perceived Intensity of Target Emotions (VAS)

Regarding VAS scores, a significant difference (F (2, 248) =3.31, *p* = 0.04, MSE = 0.34) was found for TNeutral after b1fear (Table 3). Specifically, BD I rated higher than BD II did on the target emotion (*p* = 0.04, 95% CI = 0.007~0.30). For the sake of brevity, detailed statistical results were omitted.

### 3.3. Electrocardiographic Changes

The mean RR intervals before normalization during blank were 0.79 s ± 0.10 S.D. for healthy controls, 0.80 ± 0.11 for BD I, and 0.79 ± 0.09 for BD II respectively; and they were not significantly different from each other (F (2, 259) = 0.53, *p* = 0.59, MSE = 0.006). With normalized data, we identified a significant group effect in b2fear after TNeutral (group effect, F (2, 259) = 4.68, *p* = 0.01, MSE = 0.32; emotion effect, F (3, 777) = 6.45, *p* < 0.001, MSE = 0.18; group X emotion interaction effect, F (6, 777) = 0.91, *p* = 0.48, MSE = 0.03), with the mean RR interval in controls (*p* = 0.008, 95% CI = 0.02~0.16) and BD II (*p* = 0.005, 95% CI = 0.03~0.20) longer than that in BD I (Table 4). No significant group effect was found regarding other emotional combinations. For the sake of brevity, the detailed statistical results were not reported.

### 3.4. Electrooculographic Changes

The mean eyeball movement before normalization during blank were 0.78 (mV/s) ± 0.32 S.D. for healthy controls, 0.87 ± 0.57 for BD I, and 0.83 ± 0.58 for BD II respectively, and they were not significantly different from each other either (F (2, 259) = 1.03, *p* = 0.36, MSE = 0.22). With normalized data, we identified a significant group effect in THappiness and TSadness after b1disgust (group effect, F (2, 259) = 4.44, *p* = 0.01, MSE = 0.44; emotion effect, F (3, 777) = 6.98, *p* < 0.001, MSE = 0.20; group X emotion interaction effect, F (6, 777) = 0.95, *p* = 0.46, MSE = 0.03), with the mean eyeball movement in BD I higher than that in controls (*p* = 0.045, 95% CI = 0.001~0.17) during THappiness, and the movement in BD I higher than that in BD II (*p* = 0.02, 95% CI = 0.01~0.19) during TSadness. Another significant group effect was identified for b2disgust after TNeutral (group effect, F (2, 259) = 3.56, *p* = 0.03, MSE = 0.31; emotion effect, F (3, 777) = 6.23, *p* < 0.001, MSE = 0.21; group X emotion interaction effect, F (6, 777) = 0.39, *p* = 0.89, MSE = 0.01), with the movement in BD I (*p* = 0.02, 95% CI = 0.01~0.17) higher than that in controls (Table 5). No significant group effect was found regarding other emotional combinations. Again, for the sake of brevity, detailed statistical results were not reported.

### 3.5. Relationships between Physiological Measures and Affective States

In BD I, RR interval during TSadness after b1erotica was negatively correlated with MDQ (*n* = 69, *r* = −0.35, *p* < 0.01). The eyeball movement during TSadness after b1neutral was positively correlated with MDQ (*r* = 0.36, *p* < 0.01). In BD II, RR interval during TNeutral after b1erotica was negatively correlated with HCL-32 (*n* = 54, *r* = −0.38, *p* < 0.01), and during b2neutral after TSadness was positively correlated with MDQ (*r* = 0.39, *p* < 0.01). The RR interval during b1sadness before THappiness was positively correlated with MDQ (*r* = 0.41, *p* < 0.01).

### 3.6. Summary of Differences between BD I and BD II

Compared to healthy controls, BD I and BD II were characterized differently in regard to the affective states, perceived intensity of the target emotion, and the electrocardiographic and electrooculographic changes (Table 6).

## 4. Discussion

To the best of our knowledge, this is the first trial of physiological responses to external emotions and their transitions in bipolar disorder. The RR interval in BD I was shorter (i.e., the heart rate was higher) than that in BD II during b2fear after TNeutral. The eyeball movement was quicker in BD I compared with controls during THappiness after b1disgust, and compared with BD II during TSadness after b1disgust. During b2disgust after TNeutral, BD I showed quicker eyeball movement than controls did. In BD I, the RR interval during TSadness after b1erotica was negatively correlated with MDQ. The eyeball movement during TSadness after b1neutral was positively correlated with MDQ. In BD II, the RR interval during TNeutral after b1erotica was negatively correlated with HCL-32, and that during b2neutral after TSadness was positively correlated with MDQ. The RR interval during b1sadness before THappiness was positively correlated with MDQ. Moreover, we found higher PVP in BD II than those in BD I and controls, higher MDQ in BD I than those in BD II and controls, and higher HCL-32 in both BD I and BD II than that in controls, which were consistent with previous reports [47,48,49]. The higher score of HCL-32 in BD I than that in BD II was consistent with what we have found previously [31,32,49], which might be due to the coexistence of mania and hypomania in bipolar disorder [50] and to the sufficiency of one mania episode to diagnose BD I [2].

Our first hypothesis was partly supported regarding exaggerated response to positive stimulations in BD I, but we failed to detect an increased response to negative emotions in BD II. Based on previous findings that patients with manic and mixed states considered neutral pictures pleasant [33,51,52], the accelerated heart rate in BD I compared to BD II in response to emotional transition from TNeutral to b2fear might imply that BD I patients were sympathetically more reactive when facing negative emotion fear than BD II patients did. Consistently, a higher subjective intensity of TNeutral after b1fear was found in our BD I than that in BD II.

During THappiness after b1disgust, BD I showed higher speed of eyeball movement than healthy controls did, which was in line with that the manic individuals exhibited more motoric activity in a novel environment [53]. The increased speed of eyeball movement in BD I compared with BD II during TSadness after b1disgust might be due to the depressive tendency in BD II, i.e., TSadness presented after b1disgust might initiate the depressive response, since depression is associated with diminished responses to the external emotional-stimuli [54,55]. The increased speed of eyeball movement during b2disgust after TNeutral in BD I, might again imply that BD I patients need more peace (TNeutral), and that they use more active strategies to cope with negative situations. These outcomes were consistent with that BD I relied more on active coping strategies, such as seeking professional help and stimulus reduction, while BD II relied more on negative ones, such as denying and blaming [56].

The correlation results of the current study support our second hypothesis regarding the effects of affective state on physiological parameters. In BD I, RR interval during TSadness after b1erotica was negatively correlated with MDQ, which was in line with previous reports that patients with manic and mixed states reported a higher arousal when viewing pictures [33]. It might be due to the hypersexual tendencies in BD I [43,44]. However, it remains unanswered why the correlation was specific to b1erotica. Also in BD I, the positive correlation between MDQ and eyeball movement during TSadness after b1neutral was supported by a previous study showing that the recognition of sad faces was negatively correlated with mania [16].

In BD II, the negative correlation between HCL-32 and RR interval during TNeutral after b1erotica and the positive correlation between MDQ and RR interval during b2neutral after TSadness might be connected with the attempts observed in BD II where individuals would elevate their mood by involving more sexual activities or other endeavors such as intake of food, alcohol, or drugs [57]. There was also a positive correlation between MDQ and RR interval during b1sadness before THappiness, which might be linked with a preparatory action, a phenomenon found in the movement-related cerebral potential study [58], for the anticipated THappiness. It might also imply a deficit in the processing of sadness in mania [16].

The present study suffers from several design limitations. Firstly, we used only two peripheral physiological parameters in response to the external emotions and their transitions, whether other physiological parameters were more sensitive to them remains to be seen. Secondly, each emotional combination was presented only once for one participant, repetitive presentations would help to build more reliable physiological responses. Thirdly, besides disgust, erotica, fear, happiness, neutral, and sadness, there are other emotions such as anger and surprise, which might exhibit other effects on these responses in BD I and BD II. Fourthly, although the external emotions we used were composed of pictures from the International Affective Picture System and of sounds from the International Affective Digital Sounds, the pictures were without scrambled image control [59], which might bias our results.

## 5. Conclusions

In the present study, we have demonstrated that BD I and BD II displayed different physiological responses to external emotions and their transitions, and some responses were affective state-associated. Future designs might be targeted at the cortical information processing of these external emotions and their transitions, and at the clinical management of emotion in bipolar disorder, which might eventually benefit the public safety.

## Figures and Tables

**Table 1:** Combination and permutation of external emotional stimulations after blank (lasting for 10 s).

Emotional Combination		Emotional Permutation		
Target emotion	Background emotion	*background(b1, lasting for 6 s)*	*Target(T, lasting for 8 s)*	*same background again(b2, lasting for 10 s)*
Erotica	Disgust	*b1disgust*	*TErotica*	*b2disgust*
	Fear	*b1fear*	*TErotica*	*b2fear*
	Happiness	*b1happiness*	*TErotica*	*b2happiness*
	Neutral	*b1neutral*	*TErotica*	*b2neutral*
	Sadness	*b1sadness*	*TErotica*	*b2sadness*
Happiness	Disgust	*b1disgust*	*THappiness*	*b2disgust*
	Erotica	*b1erotica*	*THappiness*	*b2erotica*
	Fear	*b1fear*	*THappiness*	*b2fear*
	Neutral	*b1neutral*	*THappiness*	*b2neutral*
	Sadness	*b1sadness*	*THappiness*	*b2sadness*
Neutral	Disgust	*b1disgust*	*TNeutral*	*b2disgust*
	Erotica	*b1erotica*	*TNeutral*	*b2erotica*
	Fear	*b1fear*	*TNeutral*	*b2fear*
	Happiness	*b1happiness*	*TNeutral*	*b2happiness*
	Sadness	*b1sadness*	*TNeutral*	*b2sadness*
Sadness	Disgust	*b1disgust*	*TSadness*	*b2disgust*
	Erotica	*b1erotica*	*TSadness*	*b2erotica*
	Fear	*b1fear*	*TSadness*	*b2fear*
	Happiness	*b1happiness*	*TSadness*	*b2happiness*
	Neutral	*b1neutral*	*TSadness*	*b2neutral*

**Table 2:** Questionnaire scale scores (mean ± S.D.) in healthy volunteers (controls, *n* = 139) and in patients with bipolar I (BD I, *n* = 69) and II (BD II, *n* = 54) disorders.

Scale	Controls	BD I	BD II
Mood Disorder Questionnaire	2.79 ± 2.04	9.41 ± 1.53 a	3.35 ± 1.88 b
Hypomanic Checklist-32	14.2 ± 5.16	22.01 ± 2.96 a	19.96 ± 2.84 a,b
Plutchik-van Praag Depression Inventory	8.83 ± 4.84	16.68 ± 12.13 a	24.65 ± 15.71 a,b

Note: a, *p* < 0.05 vs. controls; b, *p* < 0.05 vs. BD I.

**Table 3:** Intensity ratings of target emotions (mean ± S.D.; normalized, with raw data in brackets) in healthy volunteers (controls, *n* = 139) and in patients with bipolar I (BD I, *n* = 69) and II (BD II, *n* = 54) disorders.

Emotional Epoch	Controls	BD I	BD II
(b1disgust) Terotica	0.66 ± 0.32 (4.99 ± 2.29)	0.71 ± 0.27 (5.29 ± 2.02)	0.70 ± 0.31 (5.29 ± 2.56)
(b1disgust) Thappiness	0.69 ± 0.31 (5.14 ± 2.13)	0.73 ± 0.32 (5.41 ± 2.27)	0.79 ± 0.29 (5.73 ± 2.47)
(b1disgust) Tneutral	0.69 ± 0.28 (5.08 ± 2.12)	0.73 ± 0.31 (5.33 ± 2.21)	0.66 ± 0.34 (5.18 ± 2.68)
(b1disgust) TSadness	0.70 ± 0.29 (5.11 ± 2.11)	0.76 ± 0.25 (5.42 ± 1.98)	0.65 ± 0.32 (4.86 ± 2.51)
(b1erotica) THappiness	0.53 ± 0.29 (4.11 ± 2.03)	0.54 ± 0.30 (4.00 ± 2.15)	0.52 ± 0.32 (4.14 ± 2.49)
(b1erotica) TNeutral	0.51 ± 0.30 (4.07 ± 1.95)	0.58 ± 0.23 (4.36 ± 1.63)	0.50 ± 0.34 (3.94 ± 2.43)
(b1erotica) TSadness	0.48 ± 0.30 (3.90 ± 1.98)	0.49 ± 0.29 (3.94 ± 2.08)	0.52 ± 0.32 (4.06 ± 2.47)
(b1fear) TErotica	0.61 ± 0.32 (4.77 ± 2.34)	0.66 ± 0.32 (5.03 ± 2.34)	0.54 ± 0.33 (4.29 ± 2.54)
(b1fear) THappiness	0.63 ± 0.35 (4.83 ± 2.45)	0.69 ± 0.29 (5.23 ± 2.29)	0.62 ± 0.35 (4.67 ± 2.73)
(b1fear) TNeutral	0.60 ± 0.32 (4.73 ± 2.33)	0.69 ± 0.31 (5.24 ± 2.33)	0.54 ± 0.36 b (4.20 ± 2.79)
(b1fear) TSadness	0.62 ± 0.35 (4.73 ± 2.40)	0.68 ± 0.32 (5.23 ± 2.35)	0.56 ± 0.37 (4.45 ± 2.61)
(b1happiness) TErotica	0.49 ± 0.30 (3.89 ± 2.05)	0.48 ± 0.30 (3.91 ± 2.17)	0.51 ± 0.33 (4.04 ± 2.45)
(b1happiness) TNeutral	0.45 ± 0.33 (3.73 ± 2.09)	0.44 ± 0.30 (3.70 ± 2.08)	0.50 ± 0.33 (4.02 ± 2.39)
(b1happiness) TSadness	0.41 ± 0.33 (3.48 ± 2.15)	0.42 ± 0.31 (3.50 ± 2.23)	0.43 ± 0.36 (3.49 ± 2.45)
(b1neutral) TErotica	0.24 ± 0.28 (2.43 ± 1.88)	0.27 ± 0.27 (2.62 ± 1.86)	0.23 ± 0.25 (2.25 ± 1.91)
(b1neutral) THappiness	0.19 ± 0.26 (2.18 ± 1.86)	0.15 ± 0.20 (2.06 ± 1.71)	0.21 ± 0.25 (2.04 ± 1.81)
(b1neutral) TSadness	0.23 ± 0.27 (2.36 ± 1.90)	0.20 ± 0.25 (2.32 ± 2.02)	0.22 ± 0.32 (2.18 ± 2.30)
(b1sadness) TErotica	0.37 ± 0.27 (3.33 ± 1.92)	0.42 ± 0.23 (3.61 ± 1.82)	0.42 ± 0.34 (3.39 ± 2.52)
(b1sadness) THappiness	0.41 ± 0.30 (3.48 ± 2.07)	0.49 ± 0.28 (3.97 ± 2.10)	0.47 ± 0.35 (3.84 ± 2.65)
(b1sadness) TNeutral	0.38 ± 0.28 (3.38 ± 2.04)	0.43 ± 0.25 (3.62 ± 1.90)	0.47 ± 0.32 (3.71 ± 2.49)

Note: b, *p* < 0.05 vs. BD I; see Table 1 for explanations of the emotional epochs.

**Table 4:** Electrocardiogram data (mean ± S.D.; normalized) during different emotional-epochs in healthy volunteers (controls, *n* = 139) and patients with bipolar I (BD I, *n* = 69) and II (BD II, *n* = 54) disorders.

Emotional Epoch	Controls	BD I	BD II
b1disgust (TErotica)	0.56 ± 0.21	0.58 ± 0.21	0.59 ± 0.20
b1disgust (THappiness)	0.51 ± 0.22	0.52 ± 0.20	0.51 ± 0.21
b1disgust (TNeutral)	0.53 ± 0.22	0.51 ± 0.24	0.57 ± 0.18
b1disgust (TSadness)	0.52 ± 0.21	0.56 ± 0.19	0.54 ± 0.21
b1erotica (THappiness)	0.53 ± 0.21	0.52 ± 0.22	0.52 ± 0.21
b1erotica (TNeutral)	0.55 ± 0.21	0.57 ± 0.21	0.53 ± 0.21
b1erotica (TSadness)	0.56 ± 0.21	0.54 ± 0.20	0.60 ± 0.20
b1fear (TErotica)	0.58 ± 0.22	0.61 ± 0.20	0.58 ± 0.22
b1fear (THappiness)	0.53 ± 0.22	0.57 ± 0.23	0.53 ± 0.21
b1fear (TNeutral)	0.52 ± 0.22	0.52 ± 0.20	0.55 ± 0.21
b1fear (TSadness)	0.53 ± 0.20	0.53 ± 0.20	0.54 ± 0.19
b1happiness (TErotica)	0.57 ± 0.21	0.60 ± 0.20	0.62 ± 0.19
b1happiness (TNeutral)	0.57 ± 0.22	0.56 ± 0.23	0.55 ± 0.21
b1happiness (TSadness)	0.50 ± 0.22	0.57 ± 0.20	0.48 ± 0.24
b1neutral (TErotica)	0.55 ± 0.20	0.58 ± 0.23	0.54 ± 0.19
b1neutral (THappiness)	0.54 ± 0.23	0.55 ± 0.21	0.52 ± 0.17
b1neutral (TSadness)	0.55 ± 0.21	0.57 ± 0.20	0.54 ± 0.21
b1sadness (TErotica)	0.58 ± 0.24	0.56 ± 0.23	0.60 ± 0.23
b1sadness (THappiness)	0.51 ± 0.23	0.54 ± 0.23	0.52 ± 0.20
b1sadness (TNeutral)	0.57 ± 0.22	0.55 ± 0.21	0.59 ± 0.24
(b1disgust) TErotica	0.62 ± 0.18	0.61 ± 0.20	0.65 ± 0.19
(b1disgust) THappiness	0.57 ± 0.20	0.53 ± 0.24	0.56 ± 0.22
(b1disgust) TNeutral	0.57 ± 0.20	0.55 ± 0.23	0.63 ± 0.20
(b1disgust) TSadness	0.59 ± 0.21	0.56 ± 0.22	0.61 ± 0.20
(b1erotica) THappiness	0.58 ± 0.17	0.60 ± 0.21	0.58 ± 0.20
(b1erotica) TNeutral	0.61 ± 0.20	0.61 ± 0.16	0.65 ± 0.19
(b1erotica) TSadness	0.60 ± 0.20	0.62 ± 0.21	0.64 ± 0.23
(b1fear) TErotica	0.69 ± 0.21	0.69 ± 0.18	0.67 ± 0.22
(b1fear) THappiness	0.63 ± 0.21	0.64 ± 0.20	0.62 ± 0.21
(b1fear) TNeutral	0.64 ± 0.20	0.60 ± 0.20	0.65 ± 0.21
(b1fear) TSadness	0.65 ± 0.21	0.66 ± 0.21	0.66 ± 0.20
(b1happiness) TErotica	0.57 ± 0.20	0.58 ± 0.17	0.62 ± 0.18
(b1happiness) TNeutral	0.57 ± 0.19	0.55 ± 0.19	0.54 ± 0.16
(b1happiness) TSadness	0.54 ± 0.18	0.54 ± 0.19	0.53 ± 0.21
(b1neutral) TErotica	0.64 ± 0.18	0.61 ± 0.21	0.65 ± 0.18
(b1neutral) THappiness	0.55 ± 0.20	0.63 ± 0.20	0.55 ± 0.16
(b1neutral) TSadness	0.59 ± 0.20	0.58 ± 0.20	0.62 ± 0.20
(b1sadness) TErotica	0.63 ± 0.21	0.57 ± 0.22	0.61 ± 0.21
(b1sadness) THappiness	0.55 ± 0.20	0.53 ± 0.20	0.55 ± 0.19
(b1sadness) TNeutral	0.59 ± 0.20	0.59 ± 0.20	0.57 ± 0.23
(TErotica) b2disgust	0.54 ± 0.18	0.53 ± 0.21	0.58 ± 0.18
(THappiness) b2disgust	0.50 ± 0.21	0.45 ± 0.22	0.52 ± 0.21
(TNeutral) b2disgust	0.49 ± 0.20	0.49 ± 0.21	0.54 ± 0.18
(TSadness) b2disgust	0.52 ± 0.21	0.51 ± 0.21	0.55 ± 0.19
(THappiness) b2erotica	0.56 ± 0.18	0.54 ± 0.19	0.52 ± 0.18
(TNeutral) b2erotica	0.61 ± 0.19	0.58 ± 0.19	0.62 ± 0.18
(TSadness) b2erotica	0.57 ± 0.21	0.59 ± 0.20	0.60 ± 0.19
(TErotica) b2fear	0.64 ± 0.19	0.58 ± 0.20	0.63 ± 0.19
(THappiness) b2fear	0.56 ± 0.19	0.52 ± 0.20	0.56 ± 0.18
(TNeutral) b2fear	0.59 ± 0.19	0.50 ± 0.21 a	0.61 ± 0.17 b
(TSadness) b2fear	0.57 ± 0.22	0.54 ± 0.17	0.59 ± 0.21
(TErotica) b2happiness	0.58 ± 0.20	0.56 ± 0.20	0.61 ± 0.20
(TNeutral) b2happiness	0.54 ± 0.19	0.53 ± 0.19	0.49 ± 0.21
(TSadness) b2happiness	0.51 ± 0.20	0.51 ± 0.19	0.52 ± 0.21
(TErotica) b2neutral	0.61 ± 0.18	0.60 ± 0.20	0.63 ± 0.20
(THappiness) b2neutral	0.53 ± 0.20	0.55 ± 0.17	0.51 ± 0.18
(TSadness) b2neutral	0.56 ± 0.19	0.51 ± 0.18	0.56 ± 0.17
(TErotica) b2sadness	0.60 ± 0.20	0.59 ± 0.17	0.65 ± 0.21
(THappiness) b2sadness	0.52 ± 0.20	0.48 ± 0.18	0.51 ± 0.18
(TNeutral) b2sadness	0.55 ± 0.20	0.51 ± 0.22	0.54 ± 0.23

Note: a, *p* < 0.05 vs. controls; b, *p* < 0.05 vs. BD I; see Table 1 for explanations of the emotional epochs.

**Table 5:** Electrooculogram data (mean ± S.D.; normalized) during different emotional epochs in healthy volunteers (controls, *n* = 139) and patients with bipolar I (BD I, *n* = 69) and II (BD II, *n* = 54) disorders.

Emotion Epoch	Controls	BD I	BD II
b1disgust (TErotica)	0.36 ± 0.21	0.34 ± 0.21	0.36 ± 0.20
b1disgust (THappiness)	0.34 ± 0.23	0.36 ± 0.24	0.32 ± 0.19
b1disgust (TNeutral)	0.22 ± 0.19	0.28 ± 0.22	0.23 ± 0.21
b1disgust (TSadness)	0.36 ± 0.21	0.40 ± 0.20	0.38 ± 0.18
b1erotica (THappiness)	0.30 ± 0.20	0.36 ± 0.19	0.35 ± 0.22
b1erotica (TNeutral)	0.22 ± 0.17	0.26 ± 0.19	0.30 ± 0.22
b1erotica (TSadness)	0.37 ± 0.22	0.34 ± 0.19	0.39 ± 0.21
b1fear (TErotica)	0.34 ± 0.21	0.36 ± 0.21	0.42 ± 0.23
b1fear (THappiness)	0.35 ± 0.24	0.34 ± 0.22	0.31 ± 0.19
b1fear (TNeutral)	0.26 ± 0.20	0.24 ± 0.19	0.27 ± 0.19
b1fear (TSadness)	0.36 ± 0.18	0.40 ± 0.20	0.42 ± 0.19
b1happiness (TErotica)	0.37 ± 0.23	0.40 ± 0.25	0.40 ± 0.23
b1happiness (TNeutral)	0.26 ± 0.23	0.27 ± 0.22	0.24 ± 0.16
b1happiness (TSadness)	0.41 ± 0.20	0.39 ± 0.22	0.37 ± 0.24
b1neutral (TErotica)	0.34 ± 0.23	0.37 ± 0.22	0.35 ± 0.26
b1neutral (THappiness)	0.31 ± 0.20	0.34 ± 0.22	0.32 ± 0.20
b1neutral (TSadness)	0.39 ± 0.22	0.35 ± 0.19	0.40 ± 0.22
b1sadness (TErotica)	0.38 ± 0.22	0.38 ± 0.24	0.36 ± 0.19
b1sadness (THappiness)	0.32 ± 0.21	0.34 ± 0.21	0.34 ± 0.18
b1sadness (TNeutral)	0.22 ± 0.18	0.24 ± 0.20	0.31 ± 0.23
(b1disgust) TErotica	0.27 ± 0.20	0.32 ± 0.26	0.28 ± 0.19
(b1disgust) THappiness	0.30 ± 0.23	0.39 ± 0.26 a	0.30 ± 0.21
(b1disgust) TNeutral	0.25 ± 0.20	0.32 ± 0.24	0.27 ± 0.20
(b1disgust) TSadness	0.26 ± 0.21	0.31 ± 0.23	0.21 ± 0.14 b
(b1erotica) THappiness	0.33 ± 0.22	0.36 ± 0.22	0.36 ± 0.24
(b1erotica) TNeutral	0.28 ± 0.20	0.32 ± 0.20	0.33 ± 0.21
(b1erotica) TSadness	0.33 ± 0.21	0.31 ± 0.20	0.37 ± 0.23
(b1fear) TErotica	0.34 ± 0.22	0.32 ± 0.21	0.38 ± 0.20
(b1fear) THappiness	0.36 ± 0.22	0.38 ± 0.22	0.33 ± 0.18
(b1fear) TNeutral	0.31 ± 0.19	0.33 ± 0.20	0.37 ± 0.22
(b1fear) TSadness	0.33 ± 0.21	0.35 ± 0.20	0.41 ± 0.21
(b1happiness) TErotica	0.30 ± 0.19	0.31 ± 0.21	0.27 ± 0.16
(b1happiness) TNeutral	0.30 ± 0.22	0.28 ± 0.19	0.33 ± 0.25
(b1happiness) TSadness	0.30 ± 0.20	0.31 ± 0.24	0.26 ± 0.17
(b1neutral) TErotica	0.22 ± 0.19	0.21 ± 0.22	0.18 ± 0.18
(b1neutral) THappiness	0.24 ± 0.19	0.23 ± 0.18	0.28 ± 0.21
(b1neutral) TSadness	0.20 ± 0.18	0.17 ± 0.16	0.20 ± 0.15
(b1sadness) TErotica	0.35 ± 0.20	0.35 ± 0.22	0.32 ± 0.20
(b1sadness) THappiness	0.37 ± 0.21	0.34 ± 0.22	0.36 ± 0.20
(b1sadness) TNeutral	0.32 ± 0.20	0.29 ± 0.18	0.38 ± 0.24
(TErotica) b2disgust	0.33 ± 0.21	0.38 ± 0.22	0.33 ± 0.23
(THappiness) b2disgust	0.35 ± 0.22	0.39 ± 0.22	0.34 ± 0.19
(TNeutral) b2disgust	0.26 ± 0.21	0.35 ± 0.24 a	0.29 ± 0.21
(TSadness) b2disgust	0.35 ± 0.22	0.40 ± 0.23	0.35 ± 0.22
(THappiness) b2erotica	0.29 ± 0.19	0.31 ± 0.22	0.32 ± 0.24
(TNeutral) b2erotica	0.21 ± 0.16	0.24 ± 0.22	0.23 ± 0.17
(TSadness) b2erotica	0.33 ± 0.21	0.31 ± 0.22	0.33 ± 0.17
(TErotica) b2fear	0.32 ± 0.20	0.34 ± 0.24	0.38 ± 0.23
(THappiness) b2fear	0.33 ± 0.22	0.35 ± 0.22	0.31 ± 0.19
(TNeutral) b2fear	0.24 ± 0.18	0.30 ± 0.20	0.29 ± 0.18
(TSadness) b2fear	0.36 ± 0.22	0.39 ± 0.23	0.37 ± 0.19
(TErotica) b2happiness	0.34 ± 0.24	0.37 ± 0.19	0.33 ± 0.24
(TNeutral) b2happiness	0.28 ± 0.21	0.30 ± 0.21	0.32 ± 0.22
(TSadness) b2happiness	0.39 ± 0.20	0.38 ± 0.24	0.32 ± 0.22
(TErotica) b2neutral	0.29 ± 0.21	0.32 ± 0.23	0.29 ± 0.21
(THappiness) b2neutral	0.29 ± 0.20	0.30 ± 0.20	0.34 ± 0.22
(TSadness) b2neutral	0.33 ± 0.20	0.31 ± 0.21	0.31 ± 0.21
(TErotica) b2sadness	0.33 ± 0.22	0.29 ± 0.21	0.29 ± 0.18
(THappiness) b2sadness	0.32 ± 0.23	0.27 ± 0.15	0.29 ± 0.21
(TNeutral) b2sadness	0.21 ± 0.17	0.25 ± 0.22	0.28 ± 0.21

Note: a, *p* < 0.05 vs. controls; b, *p* < 0.05 vs. BD I; see Table 1 for explanations of the emotional epochs.

**Table 6:** Measurement results of affective states and perceived intensity, and of heart rate and eyeball movement in bipolar I (BD I) and II (BD II) disorders and healthy controls (controls).

Measures	Scale/Condition	BD I vs. Controls	BD II vs. Controls	BD I vs. BD II
Score	Mood Disorder Questionnaire	↑	-	↑
Score	Hypomanic Checklist-32	↑	↑	↑
Score	Plutchik-van Praag Depression Inventory	↑	↑	↓
Perceived intensity	(b1fear) TNeutral	-	-	↑
Heart rate	(TNeutral) b2fear	↑	-	↑
Eyeball movement	(b1disgust) THappiness	↑	-	-
Eyeball movement	(b1disgust) TSadness	-	-	↑
Eyeball movement	(TNeutral) b2disgust	↑	-	-

Note: see Table 1 for explanations of the emotional epochs.
